# COTIP: Cotton TILLING Platform, a Resource for Plant Improvement and Reverse Genetic Studies

**DOI:** 10.3389/fpls.2016.01863

**Published:** 2016-12-26

**Authors:** Usman Aslam, Hafiza M. N. Cheema, Sheraz Ahmad, Iqrar A. Khan, Waqas Malik, Asif A. Khan

**Affiliations:** ^1^Plant Genetic Resources Lab, Department of Plant Breeding and Genetics, University of AgricultureFaisalabad, Pakistan; ^2^Genomics Lab, Department of Plant Breeding and Genetics, Bahauddin Zakariya UniversityMultan, Pakistan

**Keywords:** *Gossypium hirsutum*, EMS mutagenesis, TILLING, point mutation, RGAs, fiber quality

## Abstract

Cotton is cultivated worldwide for its white fiber, of which around 90% is tetraploid upland cotton (*Gossypium hirsutum* L.) carrying both A and D genome. Since centuries, yield increasing efforts for the cotton crop by conventional breeding approaches have caused an extensive erosion of natural genetic variability. Mutation based improvement strategies provide an effective way of creating new allelic variations. Targeting Induced Local Lesions IN Genomes (TILLING) provides a mutation based reverse genetic strategy to create and evaluate induced genetic variability at DNA level. Here, we report development and testing of TILLING populations of allotetraploid cotton (*G. hirsutum*) for functional genomic studies and mutation based enrichment of cotton genetic resources. Seed of two cotton cultivars “PB-899 and PB-900” were mutagenized with 0.3 and 0.2% (v/v) ethyl methanesulfonate, respectively. The phenotyping of M_1_ and M_2_ populations presented numerous mutants regarding the branching pattern, leaf morphology, disease resistance, photosynthetic lesions and flower sterility. Molecular screening for point mutations was performed by TILLING PCR aided CEL1 mismatch cleavage. To estimate the mutation frequency in the mutant genomes, five gene classes were TILLed in 8000 M_2_ plants of each var. “PB-899” and “PB-900.” These include actin (*GhACT*), Pectin Methyl Esterase (*GhPME*), sucrose synthase (*GhSUS*), resistance gene analog, and defense response gene (*DRGs*). The var. PB-899 was harboring 47% higher mutation induction rate than PB-900. The highest rate of mutation frequency was identified for NAC-TF5 (EU706348) of DRGs class, ranging from 1/58 kb in PB-899 to 1/105 kb in PB-900. The mutation screening assay revealed the presence of significant proportion of induced mutations in cotton TILLING populations such as 1/153 kb and 1/326 kb in var. “PB-899” and “PB-900,” respectively. The establishment of a cotton TILLING platform (COTIP) and data obtained from the resource TILLING population suggest its effectiveness in widening the genetic bases of cotton for improvement and utilizing it for subsequent reverse genetic studies of various genes.

## Introduction

Cotton is primarily grown for its white fiber around the globe with an added advantage of its seeds as the major oil production source. Cotton belongs to the family *Malvaceae.* Its genus (*Gossypium* L.) comprises of 45 diploid species (2*n* = 2*x* = 26) and five allotetraploid species (2*n* = 4*x* = 52). *Gossypium hirsutum* L. is one of the tetraploids, possessing coverage of A (African ancestor; the *herbaceum*) and D (American ancestor; the *raimondii*) genome (Wendel and Cronn, 2003). It possesses most of the characteristic nature of both A and D genome minus the polyploidization anomalies in its 2.83 Gb nuclear genome (Grover et al., 2004; Comai, 2005; Hendrix and Stewart, 2005). According to the global econometrics, the polyploid AD genome of *G. hirsutum* contributes more than 90% to the world yarn production. Therefore, it is the best candidate in *Gossypium* genus to explore the silent variability related to its resistance mechanism and fiber quality. However, the continuous biased manipulation of available variability in cotton germplasm through conventional breeding approaches has resulted in the great loss of genetic potential added with increased vulnerability to many biological hazards. In this scenario, widening the genetic base by mutation can diversify and create novel changes in the functional genes. The mutagenesis of cotton genome can also help to have better understanding of gene function. Various mutagenesis techniques have been routinely used in gene function studies like T-DNA insertional mutagenesis (Alonso et al., 2003) and gene silencing (Wesley et al., 2001), but proved impractical, time consuming and costly in cotton species owing to gene transformation barriers. In contrast, chemical mutagenesis provides a wide range of genetic alteration options in an easy and cost effective way. Among physical and chemical mutagenic sources, EMS mutagenesis has demonstrated extensive adoption in plant improvement research programs and tested as a useful tool to activate the mute genetic potential of crop plants (Greene et al., 2003). TILLING has been proved a successful reverse genetic approach to induce and exploit genetic variation especially in crop plants. It comprises two major steps of development of EMS mutagenized population followed by detection of base pair substitutions in targeted genes. Since the invention of this dynamic reverse genetic approach in 2000 when it was first evaluated in *Arabidopsis* to develop a mutant population for gene function analysis (McCallum et al., 2000), numerous edible and cash crops including wheat (Slade et al., 2005; Uauy et al., 2009), maize (Till et al., 2004), rice (Till et al., 2007b), pea (Dalmais et al., 2008), potato (Elias et al., 2009), barley (Talamè et al., 2008), tomato (Minoia et al., 2010; Okabe et al., 2011), and sunflower (Sabetta et al., 2011; Kumar et al., 2013) have been tested and successfully TILLed in a high throughput mode for various genes and traits. However, cotton owns the most complex genetic behavior among cash crops and have been a difficult candidate to be manipulated by conventional means, and had slow rate of success. Besides, genetic engineering approaches are hindered mainly by non-availability of tissue culture in elite cotton cultivars, the cost of producing transgenic plants, behavior of the transgene and ethical concerns, associated with GMO’s. In contrast, TILLING presents a non-transgenic genetic method for the cotton genome to deal with the prevailing problems. EMS mutagenized cotton resources are not previously described for its potential application in improvement strategies and reverse genetic studies. Here, the development of EMS mutagenized TILLING populations and optimized testing of point mutations in *G. hirsutum* – var. “PB-899” and “PB-900” by CEL1 mismatch cleavage method was described. The cotton TILLING project is a first reverse genetic initiative of its kind to expand the genetic resources of upland cotton. The phenotyping related to plant growth, branching pattern and CLCuD resistance caused by EMS mutagenesis was described in M_1_ and M_2_ populations. The mutation frequency data, obtained by TILLING eight genes of various gene families in M_2_ populations, demonstrates the worth of these two resource populations and establishment of the cotton TILLING platform (COTIP). This is an enriched resource for reverse genetic analysis of various gene families and annotating the important genes, related to yield and fiber quality traits.

## Materials and Methods

### Growth Conditions of Pilot Scale TILLING Analysis

The cotton plant completes its life cycle in about 6 months, therefore, it was efficient to evaluate the selected EMS doses at seedlings stage. The SL and RL were selected as key parameters at seedling stage. Keeping growth conditions same, any changes in the SL and RL were obvious variations induced by EMS treatment. To achieve this goal, about 800 M_2_ seed samples (one boll/plant) were randomly selected from M_1_ populations of PB-899 and PB-900. The seeds were grown in a complete randomized design (CRD) in growth room on silica trays dimensioning 50 cm (L) × 30 cm (W) containing the mixture of sand: soil (1:1). The light and water conditions were kept same for all the grown seedlings. The germination rate, RL and SL was measured from 15 days old seedlings. The data for RL and SL measured in centimeter was analyzed for induced variation at seedling stage by Minitab 10.0 using Biplot analysis.

### Plant Material and EMS Treatment

For EMS treatment, 100,000 cottonseeds were de-linted with conc. H_2_SO_4_ for each of PB-899 and PB-900. The percent solution of EMS (Sigma Aldrich Cat #: M0880) @ 0.2 and 0.3% (v/v) was prepared in ddH_2_O having an electrical conductivity (EC) of 10 μscm^-1^. The EC was considered to avoid seed tissue damage from metallic ions which are usually present in non-deionized water. The de-linted seed completely dipped in aqueous solutions of EMS was kept at room temperature with continuous shaking of 50 RPM for 3 h. The mutagenized seed was washed twice with tap water, air dried and grown in the field conditions of the Postgraduate Agriculture Research Area (Faisalabad). The plant to plant and row to row distance was maintained as 30 and 90 cm, respectively. Fertilizers were applied right before sowing and pesticide treatments were applied twice at germination and squaring stage.

### Phenotyping

The M_1_ and M_2_ populations were observed for phenotypic variations in field. The phenotypic observations for leaves were made at vegetative and reproductive phases to cover all the leaf developmental stages. The branching pattern observations were recorded after appearance of first monopodial branch till maturity. The floral modifications were recorded for open flowers on similar branches on same day in comparison to non-mutagenized plants. For boll opening category, bolls were observed on daily basis from 20 days after pollination (DAP) and continued up to 40 DAP. The CLCuV tolerance was observed during 45–120 days after sowing.

### DNA Extraction, Normalization, and Pooling Setup

The young leaf tissue samples of 8000 M_2_ plants were collected at squaring stage and DNA was extracted individually using modified CTAB method. For pilot analysis, DNA extraction was performed as duplets (equally weighed tissue form two seedlings, pooled as one sample). The DNA samples were then electrophoresed on 1% agarose gel along with *Hin*dIII/λ DNA marker (Thermo Scientific, Gene Ruler 1 kb, Cat #: SM0311) and gel images were processed by “Image J 1.44p” image analysis software. The estimated concentrations were normalized to 20 ng/μl and used to prepare 4X DNA master pool plates. The normalization values (the amount of DNA from four samples to achieve accurate pooling) were obtained with the help of Harvard Hoechst DNA normalization user interphase publically available at http://dev.plasmid.med.harvard.edu/DNANORM/NormOnlyInput.jsp.

In previous TILLING studies of polyploid crops, mostly 4–6X DNA pooling approach was followed owing to their bigger genomes like wheat (*Triticum aestivum* L.) (Slade et al., 2005; Uauy et al., 2009). Similarly, for tetraploid cotton which contains far more complex genome than other cereal crops 4X DNA pooling design was followed to avoid background noise of mutations pollution. This also reduced the time taken by tedious analysis of mutation tracing in individual DNA samples. The 96 well 2.0 ml DNA plates were used for preparing Master Pool Plate. In single well of Master Pool Plate individual 4X DNA sample was prepared by combining four individual DNA samples from DNA source plate in same quantity and concentration. Each master tube in DNA source plate contained normalized DNA of one individual plant, thus, equal volumes of normalized DNA from four tubes were pooled to make 4X master pool plate. This was achieved by taking 50 μl DNA from four individual samples giving a final volume of 200 μl. The master tubes containing normalized DNA and 4X master pool plates were stored at -20°C for future use in TILLING PCR.

### CEL1 Extraction and Testing of Cleavage Activity

The seeds of celery (*Apium graveolens* L.) were grown in earthen pots and fleshy stalks from 45 days old plants were used for CEL1 enzyme extraction. The enzyme was purified in the Plant Genetic Resources Lab, PBG, UAF by standard protocol (Till et al., 2006). The activity of CEL1 enzyme was tested by two ways; (i) re-naturation of combined genomic DNA of an exotic cotton line Acala and a commercial cultivar N-78, (ii) CEL1 digestion analysis on PCR products of *NAC5* transcription factor from Acala and N-78 with using actin amplification product as internal control in digestion reaction. In the first strategy, the DNA of two genotypes were taken in equal amount, denatured at 99°C and then re-natured gradually with temp increment of -0.3°C/20 s till 65°C. The re-natured DNA was treated with a series of concentrations (Unit 1, 0.125, 0.005, 0.0028) of CEL1 enzyme, kept at 45°C for 60 min and results were analyzed on 1% agarose gel.

In the second strategy, the amplification product of two selected genes *ACTIN* and *NAC-TF* gene were used as a template from Acala and N-78. The PCR product of both genes was separately amplified from two genotypes, pooled in the pair, denatured at 99°C and re-anneal gradually for heteroduplex formation. The re-natured product was treated with CEL1 enzyme as previously described and analyzed by gel electrophoresis.

### Target Gene Amplification and CEL1 Digestion Setup

Considering low fiber quality and disease/pest epidemics on cultivated cotton in the sub-continent especially in Pakistan during last few decades, the genes related to fiber quality such as Sucrose Synthase (*GhSUS*), Pectin Methyl Esterase (*GhPME*), and disease resistance genes such as RGAs belonging to NBS-LRR class and DRGs were targeted. Cotton TILLING PCR was carried out using 96 well PCR plates in 25 μl reaction volume/sample in Thermal cycler (C1000, Bio-Rad, USA) 1.25 U of *Taq* DNA polymerase (Thermo Scientific, Cat #: EP0402), 25 ng each of forward and reverse primer, 50 mM MgCl_2_ and 5 mM dNTPs were used in 25 μl reaction volume. The PCR temperature profile followed was: initial denaturation at 95°C for 5 min, Loop 1 (eight cycles of touchdown): 94°C for 20 s, 63 to 55°C for 30 s (an increment rate of -1°C/cycle) and 72°C for 1 min, followed by Loop 2 (33 cycles): 94°C for 20 s, 50–55°C for 30–60 s depending on the gene, 72°C for 60 s (ramp to 72°C at 0.5°C/second) and a final extension step of 72°C for 5 min. These two loops were followed by denaturation step of 99°C for 7 min and Loop 3 (re-nature step of 70 cycles): 70°C for 20 s with an increment rate of 0.3°C per cycle to allow heteroduplexes formation in case of point mutation presence in the pooled DNA.

The TILLING PCR product was treated with 10 μl of CEL1 digestion mixture containing 1 μl (0.125 U) of CEL1 enzyme, 3.5 μl 10X CEL1 digestion buffer (0.1 M Tris-HCl pH 7.7, 0.5 M KCl, 0.01% Triton X-100 and 100 μM PMSF) and 5.5 μl dH_2_O in a final volume of 35 μl. The digestion reaction was carried out at 45°C for 45 min and reaction was immediately stopped by adding 10 μl 0.15 M EDTA to each well of PCR plate at 70°C for 5 min. The digested TILLING PCR mixture was added with 5 μl of DNA loading buffer (1:1, Xylene Cyanol: Bromophenol blue) and analyzed on 2.5% agarose gel containing 0.55% EtBr running in 1X TBE buffer at 100 V for 1 h. The agarose gel width was maintained to 3–4 mm thickness. The gel images were captured and analyzed on UVP Photo Doc IT 65.

The mutation frequency of the target genes was individually calculated using the formula elaborated below and cumulative estimates were recorded for both PB-899 and PB-900 M_2_ cotton TILLING populations.

$$\text{Mutation Frequency}=\frac{\text{No. of samples screened}\times \text{Genomic region studied }\left(\text{kb}\right)}{\text{No. of mutations identified}}$$

### DNA Sequencing Scheme for SNP analysis

All the 4X pooled DNA samples which were positive for SNPs were traced back for individual DNA samples in the source DNA plate for verification by sequencing. The selected samples were tested again for previously identified point mutations by gene specific amplifications using duplet DNA (2X) pooling. The samples with real mutations were sequenced by Sanger sequencing method to verify the actual mutations. The hetroduplex formation between wild type and mutant DNA amplification strands produced overlapping peaks at the mutant positions which we considered true point mutation.

## Results and Discussion

### Pilot Scale TILLING Analysis Satisfy the Use of Optimized EMS Doses

**FIGURE 1:**
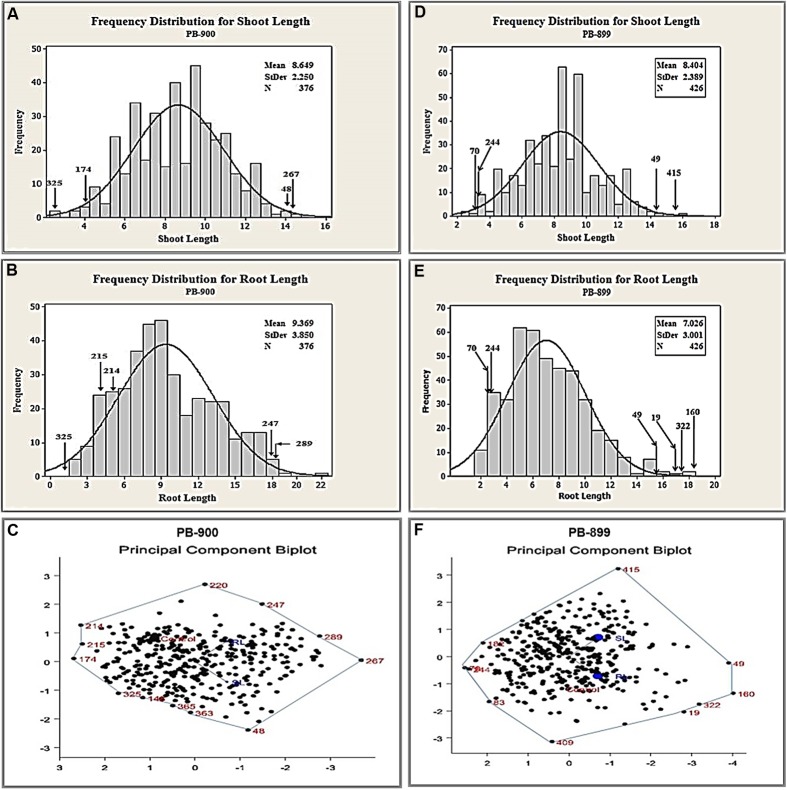
**Biplot analysis of seedling parameters to estimate genetic variability using pilot scale EMS mutagenized M_1_ cotton populations of var. “PB-899” and “PB-900.” (A)** Frequency distribution plot of M_1_ PB-900 population for SL. **(B)** Frequency distribution plot of M_1_ PB-900 population for RL. **(C)** PCB analysis of M_1_ PB-900 population. **(D)** Frequency distribution plot of M_1_ PB-899 population for SL. **(E)** Frequency distribution plot of M_1_ PB-899 population for RL. **(F)** PCB analysis of M_1_ PB-899 population.

Based on our previously described kill curve analysis of EMS mutagenesis in *Gossypium* species, 0.3% (v/v) and 0.2% (v/v) aqueous solution of EMS were estimated as effective doses for seed treatment of var. “PB-899” and “PB-900” respectively (Aslam et al., 2013). The germination percentage test used to calculate EMS LD_50_ is shown in Supplementary Figure S1. The behavior of mutagens varied between species and among cultivars within a species, therefore, to estimate the maximum likelihood of effectiveness of EMS mutagenesis, we selected two closely related cotton cultivars of cotton. The other reasons of their selection include the commercial cultivation in the region and bulk seed availability in the cotton production farm of UAF. Before the development of actual TILLING populations, a pilot scale experiment was conducted to evaluate the mutation density and effectiveness of EMS lethal doses in growth room. Root length (RL) and shoot length (SL) were observed in response of EMS doses as they are obvious indicators of tissue injury at seedling stage. Significant induced variations were observed for both genotypes (**Figures 1A,B,D–F**).

Out of 400 plants, 376 mutant plants were selected for PB-900 to see their seedling performance. In biplot analysis, the mutants scattered adjacent to vertices of the polygon, e.g., mutant no. 48 was found best fit for SL from normal distribution of wild type measurement (**Figure 1A**) and no. 247 and 289 were found best for RL (**Figure 1B**). In contrast, mutant no. 214 and 215 showed poor performance for RL. Biplot analysis also provided the information of mutants with grouped performance of RL and SL, e.g., no. 267 was found significant combine performer while no. 174 was found non-significant performer (**Figure 1C**). The frequency distribution chart also confirmed the best performing genotypes for RL and SL. Such results revealed that biplot analysis is helpful in explaining the interrelationships between individual traits and also provided an independent selection criteria based on the combined performance of several traits (Yan and Rajcan, 2002).

The angle observed for RL and SL was greater than 90° (**Figure 1C**). The trait vectors for this biplot suggested a positive correlation between the RL and SL in PB-900. The scatter plot diagram shows that mutants have significant values compared to wild type phenotype. This suggested that the EMS treatment @ 0.3% has significant induced variations.

For PB-899, 424 plants were selected to study their performance of seedling parameters, i.e., RL and SL. The angle observed for RL and SL was more than 90°. The mutants scattered near the vertices of the polygon, i.e., mutant no. 415 were found to be best fit for SL (**Figure 1D**) and no. 19, 160, and 322 were found best fit for RL (**Figure 1E**). In the PB-899 mutant no. 49 was found best for the combined performance of RL and SL (**Figure 1F**).

The significant variability in seedling traits of PB-900 and PB-899 suggested the effectiveness of EMS doses used for the development of M_1_ cotton populations.

### Development of Cotton TILLING Populations

Using tested and verified EMS doses in destructive pilot experiment, large scale cotton TILLING populations of var. “PB-899” and “PB-900” were raised at the Postgraduate Agriculture Research Station (PARS) of the Department of Plant Breeding and Genetics, University of Agriculture, Faisalabad (UAF).

**FIGURE 2:**
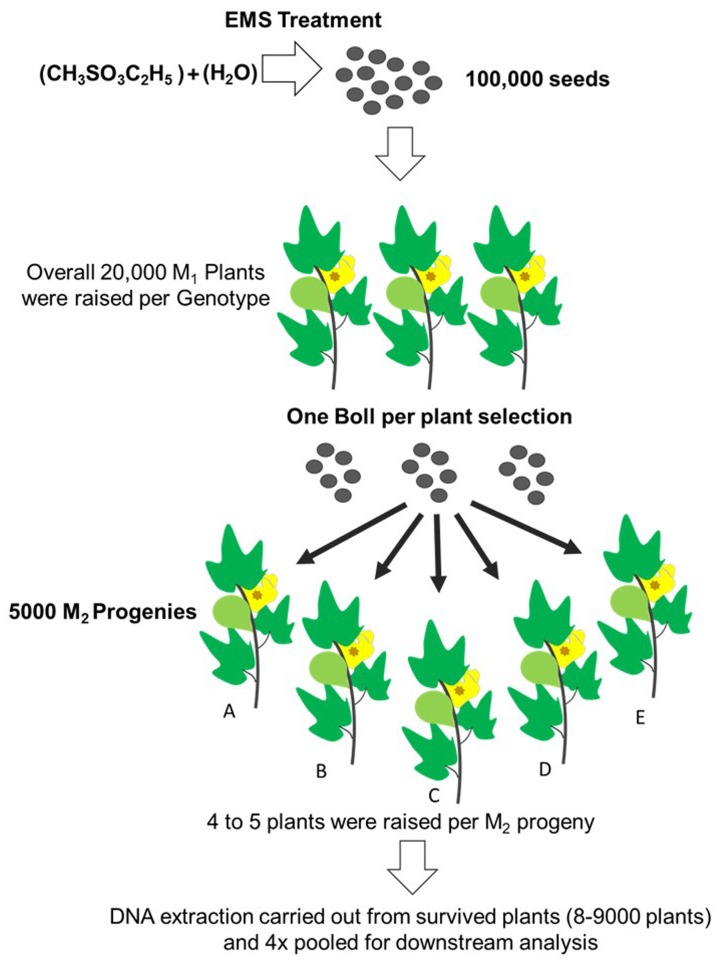
**Establishment scheme of EMS induced cotton mutant library**. 100,000 cottonseed were EMS mutagenized that produces overall on average 20,000 M_1_ plant progenies. One boll per plant selection was made from fertile plants. For each M_1_ plant, 4–5 plants were raised as a M_2_ progeny. A total of 5000 M_2_ progenies were raised. Only those plants from each progeny were tested for targeted gene TILLING which produced viable bolls. DNA was extracted from plants of each of the progeny labeled (A–E). Leftover seed was secured for future corresponding analysis and phenotyping. (*This figure was drawn by Usman Aslam*)

Initially about 100,000 cottonseeds were mutagenized with previously described 0.3 and 0.2% EMS doses for PB-899 and PB-900 respectively to raise M_1_ cotton population (Aslam et al., 2013). From almost 50% germinated plants, nearly 10% were able to produce bolls and set viable seeds. Finally, we obtained ∼5000 samples as one boll per plant to raise M_2_ progenies. Here, progeny means M_2_ plants grown from seeds of single boll harvested from M_1_ plant. Out of total M_2_ plant progenies [No. of progenies (4000) × No. of plants/progeny (5) = 20,000 × No. of seeds (4) = 80,000 expected plants], 20,000 M_2_ plants were raised for each of M_2_ PB-899 and PB-900 populations. The germination rate was observed as 55 and 60% for PB-899 and PB-900, respectively. Further mortality and sterility in M_2_ population were observed during later growth stages and lastly 8000 plants were selected for mutation screening in each of PB-899 and PB-900 population and M_3_ seeds were harvested for future analysis. During the development stages of M_1_ and M_2_ cotton populations, beginning from germination till maturity, various phenotypic observations were recorded regularly and some anomalous phenotypes were cataloged (**Figure 2**).

### Phenotyping Revealed a Diverse Range of Variant Trait Classes

**FIGURE 3:**
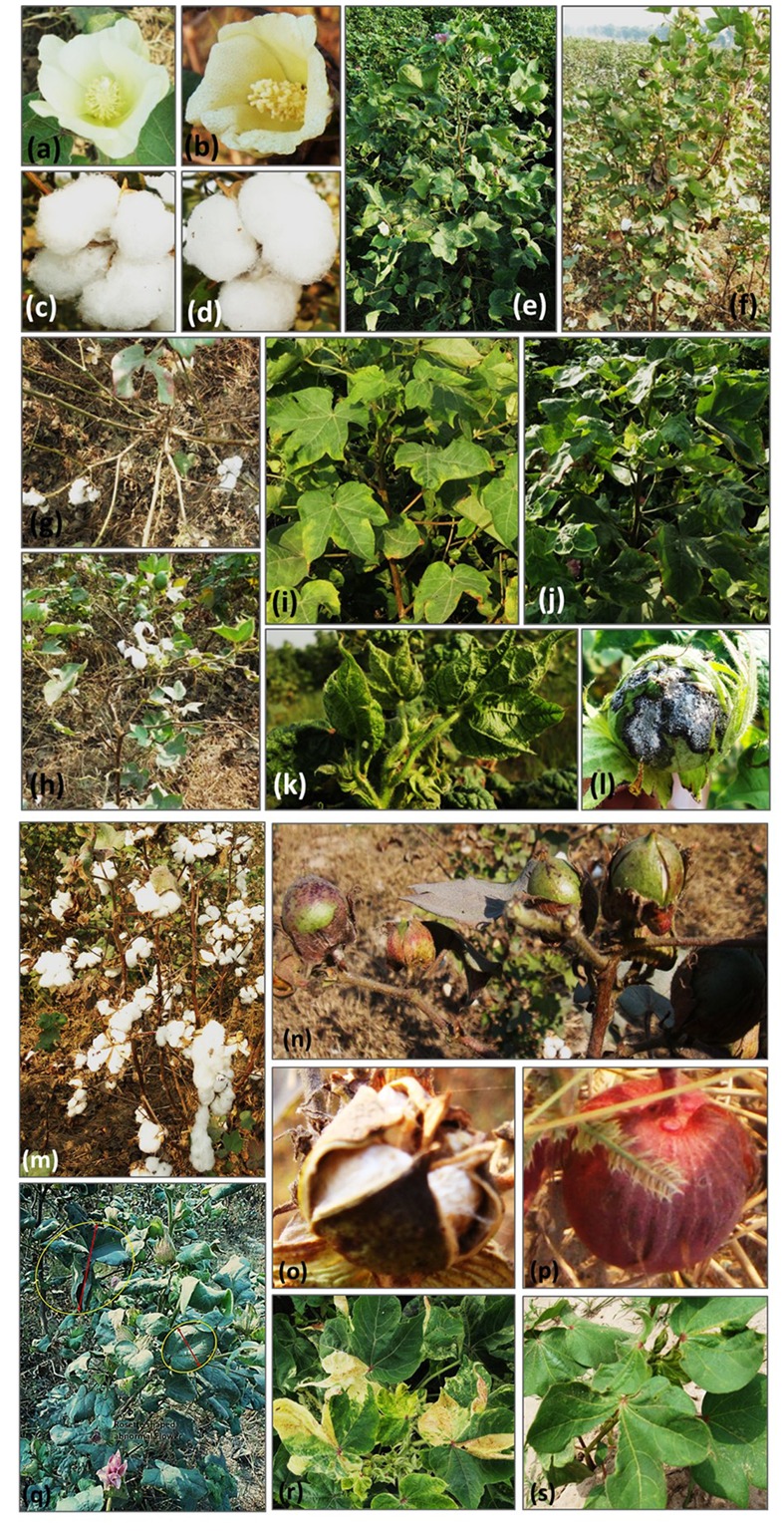
**Mutants representing various major and sub-classes of cotton phenotypes. (a)** Wild type flower of PB-899. **(b)** Mutant flower with sterile anthers. **(c)** Wild type mature cotton fruit (tetra-loculed). **(d)** Mutant cotton boll (tri-loculed). **(e)** Wild type plant of PB-899. **(f–h)** Branching pattern mutants: **(f)**; bunchy top mutant with vegetative bunch of growth at the top, **(g)**; Mutant without sympodial branches possessing bowl shaped branching pattern, **(h)**; Spiral shaped branching mutant. **(i–k)** CLCuD resistance mutants: **(i)**; CLCuV resistant mutant **(j)**; Tolerant, **(k)**; Susceptible. **(l)** Cotton boll with boll rot disease. **(m)** High yielding seed cotton mutant. **(n)** Mutant with fertile, slow developing and sterile boll development. **(o)** Mature boll development with sterile seeds. **(p)** Red colored boll mutant. **(q)** Vegetative mutant showing palm shaped large leaf development and abnormal rosette shaped flower, the resultant boll had sterile seeds. **(r)** Variegated mutant with leaf lesions. **(s)** Irregular lobe symmetry of leaf. (*This figure was designed by Usman Aslam and all the photographs included in it were also taken by Usman Aslam.*)

In cotton TILLING platform, the phenotypic variability was observed both in M_1_ and M_2_ populations of PB-899 and PB-900. Numerous phenotypes were recorded in both cultivars possessing genetic lesions. Seven traits were observed which showed several variant classes. The most number of variable traits were observed in plant stature and leaf shape, each of which are sub-categorized into four classes (**Table 1**.). The most commonly observed phenotypes were related to plant stature, fruit type and CLCuV disease resistance (**Table 1**). Some examples of the mutated phenotypes correspond to the branching pattern, flower sterility, boll maturity and CLCuV tolerance can be seen in **Figure 3**. The CLCuV tolerance was estimated by characteristic leaf symptoms of the virus infection shown in **Figure 3k**.

**Table 1:** Occurrence of induced phenotypic variation in important traits of the mutant populations.

Trait	Variation	No. of M_2_ plants
1 Branching pattern	Spiral shaped	4
	Bowl shaped	15
2 Plant stature	Bunchy top	3
	Umbrella shaped	7
	Dwarf	157
	Tall	8
3 Boll Type and shape	Oval tiny	33
	Green big	6
	Purple color	1
4 Leaf shape	Large palm	8
	Fuse lobed shaped	113
	Variegated	3
	Irregular lobe symmetry	7
5 Fruit type	Un-opened mature boll	340
	Tri-loculed	730
	Opened infertile bolls	870
6 Anther	Inflated hollow	22
	Infertile	56
7 CLCuD tolerance	Partial resistant	230
	Resistant	86
	Susceptible	150

### Gene CODDLE Analysis and the Primer Designing

**FIGURE 4:**
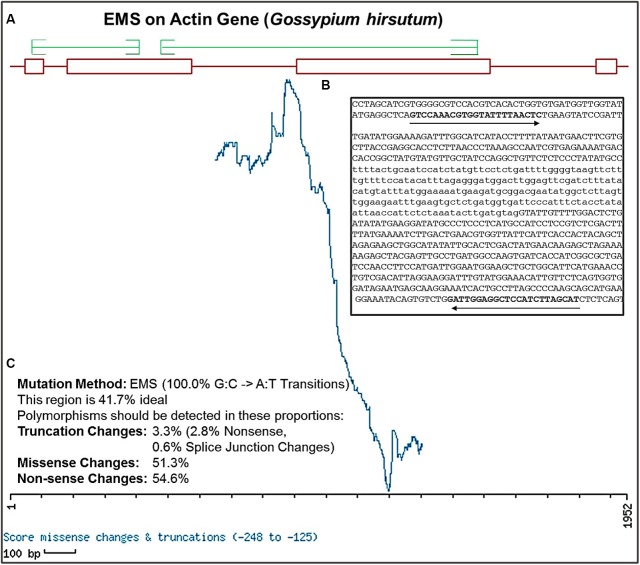
**Selection of effective regions for EMS mutation induction and primer designing using CODDLE. (A)** Output of CODDLE program showing regions of target gene *GhACTIN* with maximum EMS mutagenesis effectiveness. Red boxes indicate exons and lines indicate introns, green lines are positions selected for primers designing. **(B)** Position of primer pair in gene sequence calculated by CODDLE. **(C)** Percent estimates of different mutation types in the selected region produced by EMS induced mutation method.

To design highly specific and efficient primer pairs for target genes, nucleotide sequences of genomic DNA and CDS were retrieved from the NCBI database. To determine the effective region of target genes with maximum likelihood to be functionally altered by EMS mutagenesis, we use web accessible tool CODDLE^1^ for sequence analysis. Several isoforms of each gene were aligned, their sequences were analyzed for conserved blocks and primers were designed by PRIMER3 accordingly covering the CODDLE approved functionally affective gene regions. Various primer set were designed for each gene during the cotton genetic screen (Supplementary Table S1). EMS induces GC to AT transition mutations and CODDLE process this information using the respective CDS statement of the gene to analyze the input genomic region of a target gene. Based on the selected amplicon window size, the CODDLE scoring matrix calculation generated a graph depicting the probable effectively mutagenized region with an expected mutation rate of all possible mutation kinds such as truncation, missense and non-sense or silent mutations (**Figure 4**). The **Figure 4** demonstrates the CODDLE analyzed the region of *G. hirsutum* actin gene (*GhACT*). The highest peak values of *ACTIN* window scoring matrix are the regions of maximum likelihood of desirable alterations. The genes possessing larger introns were processed in smaller sections to cover the maximum exonic regions and primers were designed in the introns. To confirm the primer efficacy for single and pooled DNA samples, the target genes were amplified using the respective DNA samples and their amplicons were analyzed by gel electrophoresis.

### Standardization of DNA Extraction Procedure and Pooling Set Up

**FIGURE 5:**
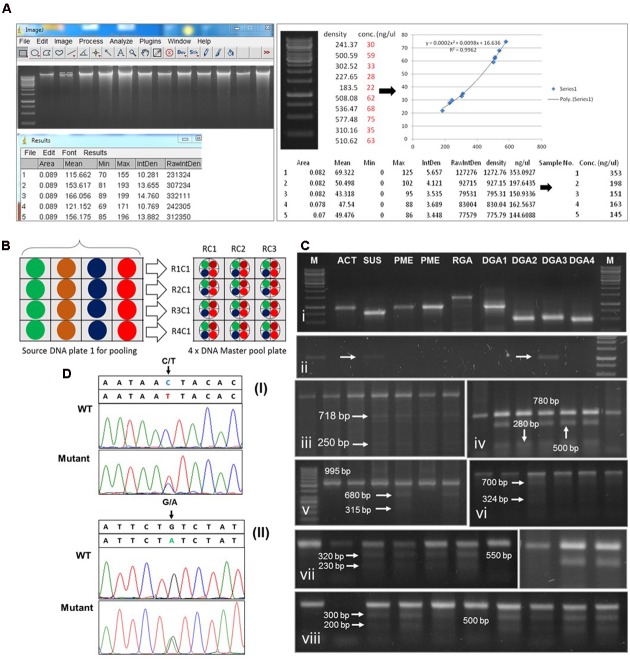
**Schematic representation of cotton TILLING results. (A)** Estimation of DNA concentration by Image J software and normalization. Left part showing band intensity measurement, right part is showing construction of reference equation (second order polynomial) and DNA normalization. **(B)** Schematic diagram of 4X DNA pooling plan. Each color is indicating individual DNA samples in a single column. R1C1 to R1C4 in the left side is indicating the code name of individual pool sample in master pool plate. RC1 in the upper side is the code name of 4X pooled DNA from single row of source DNA plate. Normalized equal concentrations are indicated by four small circles a big circle. Color is indicating respective samples in a row. **(C)** Agarose gel analysis of point mutations detection in TILLed genes. **(C-i)** Optimization of TILLING PCR on normalized DNA stored in 1xTE buffer. **(C-ii)** PCR amplification from DNA after 3 month storage in ddH_2_O. arrows indicate weak amplification **(C-iii)** Mutation detection by CELI mismatch cleavage assay in **(iii)** *GhACT* gene, **(C-iv)** *GhSUS*, **(C-v)** *GhPME*, **(C-vi)** *GhRGA* **(C-vii,viii)** *GhDRGs;* first lane is in each gel image indicate wild type amplicon band and mutant cleavage fragments are pointed by arrows and respective size. TILLING PCR product was digested with CELI enzyme and electrophoresed on 1–3% agarose gel depending on the size of digested PCR products. **(D)** Verification of point mutations by sequencing analysis. **(i)** C to T substitution is indicated by an arrow in GhMIC-3 sequence analysis. WT indicate wild type sequence with no sequence error, mutant indicate the mutated sequence **(ii)** G to A substitution is indicated by an arrow in *GhCHI* sequence analysis. WT indicate wild type sequence without mutation, mutant indicate presence of point mutation in the sequence.

Normalization and selection of appropriate DNA pooling level has a direct impact on the efficiency of mutation screening in huge populations which also have an ultimate link with DNA quality, therefore, to avoid erroneous and false positive results in downstream TILLING PCR analysis, good quality DNA is a crucial requirement. Young green leaves were used for DNA isolation from M_2_ plants as older leaves contain high phenolic compounds and polysaccharides. The DNA of individual plant samples were extracted using the modified CTAB method. To obtain pure and good quality DNA, traces of phenolics and polysaccharides were removed using 2% β-mecrcaptoethanol in CTAB. To avoid DNA hydrolysis during long storage, precipitated DNA was washed with absolute alcohol instead of 70% ethanol and DNA was stored in 1X TE buffer (10 mM Tris-HCl, pH 7.5, 1 mM EDTA pH 8.0) added with 3.2 μg/ml RNase,) instead of dH_2_O. The long storage of DNA in dH_2_O was observed to have degradation and shearing within 3 to 5 months and show dead PCR amplifications. In comparison, DNA storage in 1X TE buffer allows long term integrity and stability even at 4°C (**Figures 5A,Ci,ii**). For DNA quality and quantity estimates we used “Image J” interphase to analyze agarose gel images of DNA samples. It generates estimates of accurate DNA concentration of samples using the second order polynomial equation constructed from the reference band intensity of *Hin*dIII/λ DNA marker run along with DNA samples (**Figure 5A**).

Accurate and efficient DNA pooling is the key to success in TILLING and required to avoid high signal to noise ratio in PCR amplifications from pooled samples. Effective pooling can only be achieved if each individual DNA would be presented in nearly the same concentration in each sample. This was attained by normalization of DNA concentrations of all samples to one standard concentration, e.g., 20 ng/μl in this case. The amount of genomic DNA used per reaction was 50–80 ng depending upon the primer workability. The 4X DNA pooling approach was followed to avoid signal to noise ratio of background false positives because of large genome of tetraploid cotton (**Figure 5B**). To achieve this goal, Hoechst DNA normalization interphase^2^ was used that serves the best to get accurate estimates of concentration for normalization purposes. The example file (Excel format) showing normalized concentrations of M_2_ DNA samples (used to design master pool plate) and relative information about DNA and H_2_O/TE buffer volume is demonstrated in the **Figure 5A**, right panel.

### Testing of CEL1 Mismatch Cleavage Activity

The most crucial and difficult step in mutation analysis is the identifications of particular site of point mutations. The mismatch specificity of CEL1 has enabled us the development of highly effective and user-friendly mutation detection methodology. The CEL1 enzyme is named after its source plant celery (*A. graveolens* L.) a close relative of Ajwain, as both belonging to the parsley family *Apiaceae*. It possesses a novel endonuclease CEL1 having molecular weight of 43 kDa that can detect destabilized regions of double stranded DNA helices, such as mismatches, insertions and deletions and most specifically base pair substitutions (Pimkin et al., 2007). To deal with such a large scale screening for mutation identification, CEL1 extraction protocol was established at the Plant Genetic Resource Lab, PBG, UAF, Pakistan (Supplementary Figure S2). To verify the accurate extraction of CEL1 endonuclease, testing of its precise mismatch activity and standardizing its working concentration for cleavage assay, two strategies were followed.

In one scheme, genomic DNA of two cotton cultivars with different genetic makeup were used, considering that they may possess huge number of SNPs compared to each other. The genomic DNA of an exotic upland cotton genotype Acala and a commercial mutant cultivar “NIAB-78” were taken in equal amount, denatured at high temperature and then re-natured gradually to low temperature with the principal that both the genotypes being the tetraploid having nearly similar genome with SNPs and deletions/additions at countless sites. When the re-natured DNA was treated with CEL1 digestion mixture it showed light smear on agarose gel indicating DNA cleavage compared to control DNA (re-natured mixture of Acala and NIAB-78 genomic DNA without CEL1 treatment) showed bright long smear intense near higher molecular mass position (Supplementary Figure S3A). This indicated the presence of numerous dissimilarities of genetic makeup leading to inefficient re-annealing.

In second scheme, PCR products of *NAC*-Transcription Factor was used as template in CEL1 digestion assay that considered to have SNP in mutant cotton cultivar NIAB-78 compared to exotic cultivar Acala which was considered abiotic stress susceptible genotype. Additionally, PCR product of a house keeping gene “actin” was used as an internal in digestion assay. The actin gene revealed no SNPs giving no restriction bands in CEL1 treatment, however, in case of *NAC-TF* gene, the re-natured product showed multiple bands after CEL1 treatment as compared to control treatments indicating SNPs at this locus in two genotypes (Supplementary Figures 3B,C). Multiple concentrations were tested for mutation detection in cotton TILLING populations. Each concentration was observed effective in DNA digestion but, 0.125 and 0.02 U showed higher rate of digestion. Moderate digestion was observed with 0.005 and 0.008 Units. In case of PCR product digestion analysis, all treatments showed similar digestion activity, which is the indication of good efficiency of CEL1 endonuclease. In cotton TILLING analysis, 0.005 units of CEL1 extract was used per 25 μL reaction for point mutation detection to avoid false positives (Supplementary Figure S3).

### SNP Analysis and Mutation Frequency Estimation in Cotton TILLING Populations

The chemical mutagens are effective sources of mutation induction and widely used in reverse genetic studies. The EMS induces GC = AT transitions mutations in target organisms (Till et al., 2004, 2007a). In our experiment, the induced point mutation frequency was estimated by TILLING of eight genes on M_2_ cotton populations of PB-899 and PB-900 using agarose gel aided CEL1 mismatch cleavage detection system (Raghavan et al., 2007).

The genes screened for point mutations analysis in *G. hirsutum* var. “PB-899” and “PB-900” include actin (*GhACT*), Pectin Methyl Esterase (*GhPME*), sucrose synthase (*GhSUS*), NBS-LRR *RGAs* and *DRGs*. The *GhPME* and *GhSUS* play key role in fiber elongation in cotton. Pectin is a major component of fiber related primary cell wall and pectin modification enzymes such as PME help regulate fiber elongation. In longer fiber chromosome introgressed lines (CSILs), *PME* was found upregulated during early stages of fiber development (Fang et al., 2014). Similarly, sucrose is a form of sugar molecule which is involved in synthesis of cell wall precursors and also required for cell homeostasis. Sucrose synthase is a major sucrolytic enzyme which reversibly converts sucrose into fructose and UDP-Glucose in plants (Geigenberger and Stitt, 1993). SUS plays important role in fiber development and specifically localizes in fiber initials (Ruan and Chourey, 1998; Ruan et al., 2003). Keeping in view these facts, we hypothesize that TILLING of *PME* and *SUS* genes could be more probable to produce fiber related mutants. In disease resistance category, RGAs are key markers for *R*-genes (disease resistance) that play important roles in disease resistance mechanisms and are conserved among plant species, predominantly with NBS-LRR domain which constitutes largest R gene family (Khan et al., 2016). In DRGs class, total four genes were tested, among them, two were pathogen related [Chitinase Gene (*GhCHI*) and meloidogyne-induced cotton-3 gene (*GhMIC-3*)] and two abiotic stress associated [class III peroxidase (*Ghpod5*) and NAC transcription factor 5 (*GhNAC5*)]. The *GhNAC5* was screened in two sections, i.e., *GhNAC5-1* and *GhNAC5-2*. Out of eight genes, *GhACT, GhSUS* and all members of *GhDRGs* were more prone to EMS induced point mutation while *GhPME* and *GhNBS-LRR-RGAs* showed least mutation rate, possibly due to out of reach of the mutation induction hot spots in cotton genome (**Figure 5C**; **Table 2**). In the M_2_ genetic screen of *G. hirsutum*, numerous mutants were identified, which showed the success of TILLING approach in the creation of genetic variability in tetraploid genomes.

**Table 2:** Mutation frequency estimation in EMS mutagenized cotton populations of var. “PB-899” and “PB-900.”

Sr. #	Name of target tilled genes	Gene locus number	Genome size per gene (bp)	Number of M_2_ progenies screened	No. of mutations identified (PB-899)	Mutation frequency (PB-899)	No. of mutations identified (PB-900)	Mutation frequency (PB-900)
1	Actin (*GhAct*)	AF059484	968	8,000	43	1/180 kb	30	1/258 kb
2	Sucrose synthase (*GhSuS*)	FB742816	780	8,000	33	1/189 kb	18	1/346 kb
3	Pectin methyl esterase (*GhPME*)	JX003001.1	995	8,000	3	1/2.65 mb	2	1/3.9 mb
4	Resistance gene analogs (*GhRGAs*)	AY627695	1000	8,000	4	1/2.0 mb	0	-
5	Defense response genes (*GhDRGs*)							
I	*Gossypium hirsutum* class III peroxidase (*Ghpod5*)	AF485267	924	8,000	49	1/150 kb	14	1/528 kb
II	*G. hirsutum* chitinase gene (*GhCHI*)	Z68152	974	8,000	51	1/153 kb	17	1/458 kb
III	*GhNAC5* (*G. hirsutum* NAC domain protein (NAC5)	EU706348	1112	8,000	151	1/58 kb	84	1/105 kb
V	*GhMIC-3* (*G. hirsutum* meloidogyne-induced cotton-3 gene)	GQ231919	511	8,000	45	1/91 kb	13	1/314 kb
	Total		7264		379	1/153 kb	178	1/326 kb

The defense related gene *GhNAC5* showed the highest mutation density per unit genome in both M_2_ cotton TILLING populations (**Figures 5Cvii,viii**; **Table 2**), however, the cumulative mutation frequency was estimated as one mutation per 153 and 326 kb for PB-899 and PB-900, respectively, which is higher than Sunflower (Sabetta et al., 2011; Kumar et al., 2013). The PB-899 showed 1.5–3.5 fold higher mutation density than other crop species, for example rice, pea, tomato, soybean and maize which possess diploid genomes perhaps this frequency figure is far less than that of other polyploid genomes such as wheat and brassica (Till et al., 2004, 2007b; Cooper et al., 2008; Uauy et al., 2009; Stephenson et al., 2010; Knoll et al., 2011; Chen et al., 2012). This variable response of two different cotton cultivars might be dose dependent or could be due to the differences in their genetic makeup. Because the sterile plants were not used in mutation analysis, this could also be the result of the EMS characteristic effect of sterility (Greene et al., 2003; Perry et al., 2003) that make a plant species more vulnerable to lethal mutations resulted in a higher rate of infertility, i.e., 22% in M_2_ of PB-900 (**Table 3**). These results suggested that EMS dose is not only species specific but also genotype specific and depends upon mutagenic conditions and type of plant materials used for mutagenesis. These results also suggest rough estimates of *G. hirsutum* strength to withstand particular doses of EMS due to multiple gene redundancies in its tetraploid genome (Dalmais et al., 2008).

**Table 3:** Growth effects of EMS mutagenesis on var. PB-899 and PB-900 cultivars.

Calculated EMS dose to develop TILLING population	0% EMS M_0_ seed	0.3% (v/v) [PB-899]	0.2% (v/v)-[PB-900]
M_1_ seeds grown in the field	1000 each	100,000	100,000
Percentage of germinated M_1_ plants	80% (PB-899), 75% (PB-900	45%	55%
Percentage of plants having viable fertile bolls	80% (PB-899), 75% (PB-900	10%	13%
Number of M_2_ plants grown	800 each, [boll descent progenies]	20,000	20,000
Percentage of germinated M_2_ plants	85% (PB-899), 75% (PB-900), [boll descent progenies]	55%	60%
Percentage of plants produced infertile bolls	0%	15%	22%

The actin is one of the universal housekeeping gene in plants and animals. It plays a crucial role in the plant cytoskeleton dynamics and carryout various processes in cell division, cell expansion and cell shape maintenance (Staiger and Schliwa, 1987; Staiger and Cande, 1991). The purpose of targeting actin was to estimate the effectiveness of EMS lethality in a constitutive gene expression of conserved genetic pathways in the cotton plant. In cotton genetic screen, a total of 43 point, mutations were identified in PB-899 cotton genotype for actin while 30 alterations were detected in PB-900 for *GhACT* (**Table 2**). The higher mutation rate in PB-899, i.e., 1 mutation/180 kb than that of PB-900 (1/258 kb) suggested higher penetration of EMS induced variability in PB-899, however, PB-900 was observed less prone to mutation induction. The overall data suggested that the effectiveness of EMS mutagenesis is not only species dependent but also genotype dependent within a single species (Aslam et al., 2013).

*GhPME* showed least alterations in response to EMS mutation induction. In M_2_ genetic screen of cotton, only three point mutations were identified in PB-899 and 2 in PB-900 cotton genotypes. Similar results were found for cotton RGAs (**Table 2**). In DRG class, all genes showed considerable mutation density with *NAC-5* presenting highest mutation frequency in var. “PB-899,” i.e., 1 mutation/58 kb. These results coincide with the phenotypic performance of two cultivars which presented considerable variations in CLCuD incidence level, suggesting the possible role of *NAC-5* against CLCuV resistance in cotton.

The point mutations detected by CELI mismatch cleavage assay were confirmed by re-sequencing. The DNA samples for which mutations were identified were 2X pooled, PCR amplified and after verifying by CELI cleavage, samples were used for sequencing. In sequencing data, the overlapping signal peaks with base pair substitution verifies the actual mutation site (**Figure 5D**). Overall, the mutation density data of five gene classes per genotype demonstrates the success rate of development of these resource TILLING populations, however, due to a limited number of genes selection for point mutation analysis, further screening will help to reveal mutation enrichment in cotton TILLING populations.

### Seed Maintenance

The seeds of PB-899 and PB-900 plants were harvested from M_1_ populations in one boll/plant fashion. The harvested bolls were ginned and cottonseeds were packed in craft paper seed storage bags with numerical numberings for example M_1_-PB-899-1, M_1_-PB-899-2, M_1_-PB-899-3, M_1_-PB-899-4, and so on. Similarly for PB-900, M_1_-PB-900-1, M_1_-PB-900-2, M_1_-PB-900-3, M_1_-PB-900-4, and so on. The M_2_ plants were raised from seeds of single boll/plant in boll to row progenies fashion. The harvested seed was packed in craft paper seed storage bags and labeled as following.

M_2_-PB-899-1a, M_2_-PB-899-1b, M_2_-PB-899-1c, M_2_-PB-899-1d. M_2_-PB-899-2a, M_2_-PB-899-2b, M_2_-PB-899-2c, M_2_-PB-899-2d, M_2_-PB-899-3a, M_2_-PB-899-3b, M_1_-PB-899-3c, and so on. Here, numeral 1, 2, 3 represents progeny number and small alphabets a, b, c, d represents plants in single progeny. The M_1_ and M_2_ seeds packages were deposited in seed storage facility (Temperature: 4°C, humidity < 30% without light) of Department of Plant Breeding and Genetics, University of Agriculture Faisalabad (UAF), Pakistan.

## Conclusion

The need for crop improvement to combat with biotic and abiotic problems in agriculture is increasing with the changing environmental perspectives. TILLING is a useful reverse genetic tool, used to create and identify mutations in various organisms especially in plants and successfully manipulated in several crop species. Furthermore, as EMS generates an allelic series of targeted genes, it is possible to explore the role of desired genes that are otherwise not likely to be recovered in genetic screens, based on insertional mutagenesis/transformation. Cotton is the most important fiber crop and the pillar of Pakistan’s economy. It is combating a long list of diseases and physiological problems, right from the germination to maturity, that endanger its yield to a great extent. Although, the conventional breeding approaches contributed a lot to cotton improvement, but simultaneously it also resulted in narrowing down of the genetic background and elimination of useful qualitative and quantitative alleles from cotton. Therefore, the creation of a new allelic resource population for crop improvement is vital. The cotton TILLING is the very first step in Pakistan taking conventional mutagenesis to more advanced and sophisticated form and open the new doors to perform gene function analysis. The TILLING analysis of cotton M_2_ population at a large scale represents the effective induction of point mutations in studied genomic region. Out of eight genes tested in cotton, DRGs found more effective to mutagenesis by EMS. This could be due to the presence of DRG locus in mutation hot spot regions of cotton genome. The higher mutation rate in DRGs suggested the usefulness of cotton TILLING populations in disease resistance research especially for CLCuD. TILLING analysis was performed on very little genomic region of cotton, therefore further screening of more genes is in progress. With the availability of these resource populations to cotton breeders and researchers, we are hopeful to prove its worth in cotton plant improvement research.

### Future Perspectives

We are working on the development of *in silico* database for all population individuals, which is necessary for all possible collection of mutants. It will cover the recording of all types of plant morphologies appeared in M_3_ population. This all data will be stored in a web accessible cotton TILLING database for information about mutant types. Secondly, expression behavior of mutated genes at the whole plant level will be performed to identify potential mutants with desirable traits that can be used in ideotype breeding. Furthermore, the developed TILLING populations will be screened for abiotic stress related genes in an effort of uncovering the left over induced variability.

## Author Contributions

The research is conducted, planned and supervised by Project Investigator AAK and Co-PI HMNC. UA optimized EMS mutagenic doses, perform the cotton seed mutagenesis, look after the M_1_ and M_2_ field populations and conducted the phenotyping. The pilot experiment was performed by SA, DNA extraction, TILLING PCR and CEL1 digestion assay was optimized and performed by UA. Data analysis was done by UA. WM helps in layout and management of field and lab experiments. The manuscript was written by UA, contributed by AAK and HMNC. Figures and tables were taken and designed by UA, biplot graphs were designed by SA. Logistics were provided by IAK.

## Conflict of Interest Statement

The authors declare that the research was conducted in the absence of any commercial or financial relationships that could be construed as a potential conflict of interest. The reviewer BC and handling Editor declared their shared affiliation, and the handling Editor states that the process nevertheless met the standards of a fair and objective review.

## Footnotes

1 http://www.proweb.org/coddle

2 http://dev.plasmid.med.harvard.edu/DNANORM/NormOnlyInput.jsp

## Abbreviations

CLCuD: cotton leaf curl disease
CLCuV: cotton leaf curl virus
cm: centimeter
CODDLE: codons optimized to discover deleterious lesions
CTAB: cetyl trimethylammonium bromide
ddH_2_O: double distilled deionized water
DRGs: defense response genes
EMS: ethyl methanesulfonate
Gb: giga base pairs
kb: kilo bases
NGS: next generation sequencing
RGAs: resistance gene analogs
RL: root length
SNP: single nucleotide polymorphism
SL: shoot length
TILLING: Targeting Induced Local Lesion IN Genomes
