# Supplementary material for: COTIP: Cotton TILLING Platform, a Resource for Plant Improvement and Reverse Genetic Studies
Source: Front Plant Sci. 2016 Dec 26;7:1863. doi: 10.3389/fpls.2016.01863 (PMC5183611; doi:10.3389/fpls.2016.01863)
Supplement: Supplementary file 1 [file Table_1.PDF]

Supplementary Table 1. List of primers used for amplification of target genes in cotton TILLING populations.

Sr. #	Gene Name	Primer Name	Primer Sequence
1	Actin (*GhAct*)	Act2-TL-F	TTTTGGACTCTGGAGATGGG
		Act2-TL-R	ATCGTACTCCGCCTTAGCAA
2	Sucrose Synthase (*GhSuS*)	SS1-TL-F	ATGGCTTCAATCAGTGTTTGTG
		SS1-TL-R	CTTCTGGCAGTGAATTTGGA
3	Resistance Gene Analogues (*GhRGAs*)	RGA1-TL-F	TAGAAAGGTCAAGGGCAAGG
		RGA1-TL-R	AGTGGCAATCCTGCACATTT
		RGA2-TL-F	ACAAGTTCAGCACCACACCA
		RGA2-TL-R	CACCGCACCTTTCAGCTACT
		RGA3-TL-F	CTTGAAAGCAAGGGGGAAG
		RGA3-TL-R	GAGGGGTGGGTAAGACAACA
4	Pectin Methyl Esterase (*GhPME*)	PME1-TL-F	TCATGTAGCTCCACGTTGTACC
		PME1-TL-R	GGTATGCTAAAATGCCACACCT
Defense Response Genes (*GhDRGs*)			
5	Class Ⅲ peroxidase *(POD 6)*	DG1-TL-F	TTTGCCATTGGAGTTGTTCA
		DG1-TL-R	AGCTGTACAAACACGCCTGA
6	Gh Chitinase gene (*GhCHI*)	DG2-TL-F	GCCCCACACGTAAAACACTT
		DG2-TL-R	GTTGGATCCTTTGCCACATT
7	*(NAC5)*	DG3-TL-F	GTTTGCCTCCAGGGTTTAGG
		DG3-TL-R	CCCTCATGGAGGAATCTTTG
		DG4-TL-F	GGTTCTTTGCCGGATCTACA
		DG4-TL-R	TCCCTGGAACTGAATGTGAG
8	*MIC* (Melydogyne induced defense gene)	DG5-TL-F	ATGGCTTGTCCTCCAACTCA
		DG5-TL-R	GAGGCAGGTTAATTGCAACC
