# Supplementary material for: COTIP: Cotton TILLING Platform, a Resource for Plant Improvement and Reverse Genetic Studies
Source: Front Plant Sci. 2016 Dec 26;7:1863. doi: 10.3389/fpls.2016.01863 (PMC5183611; doi:10.3389/fpls.2016.01863)
Supplement: Supplementary file 2 [file Presentation_1.PDF]

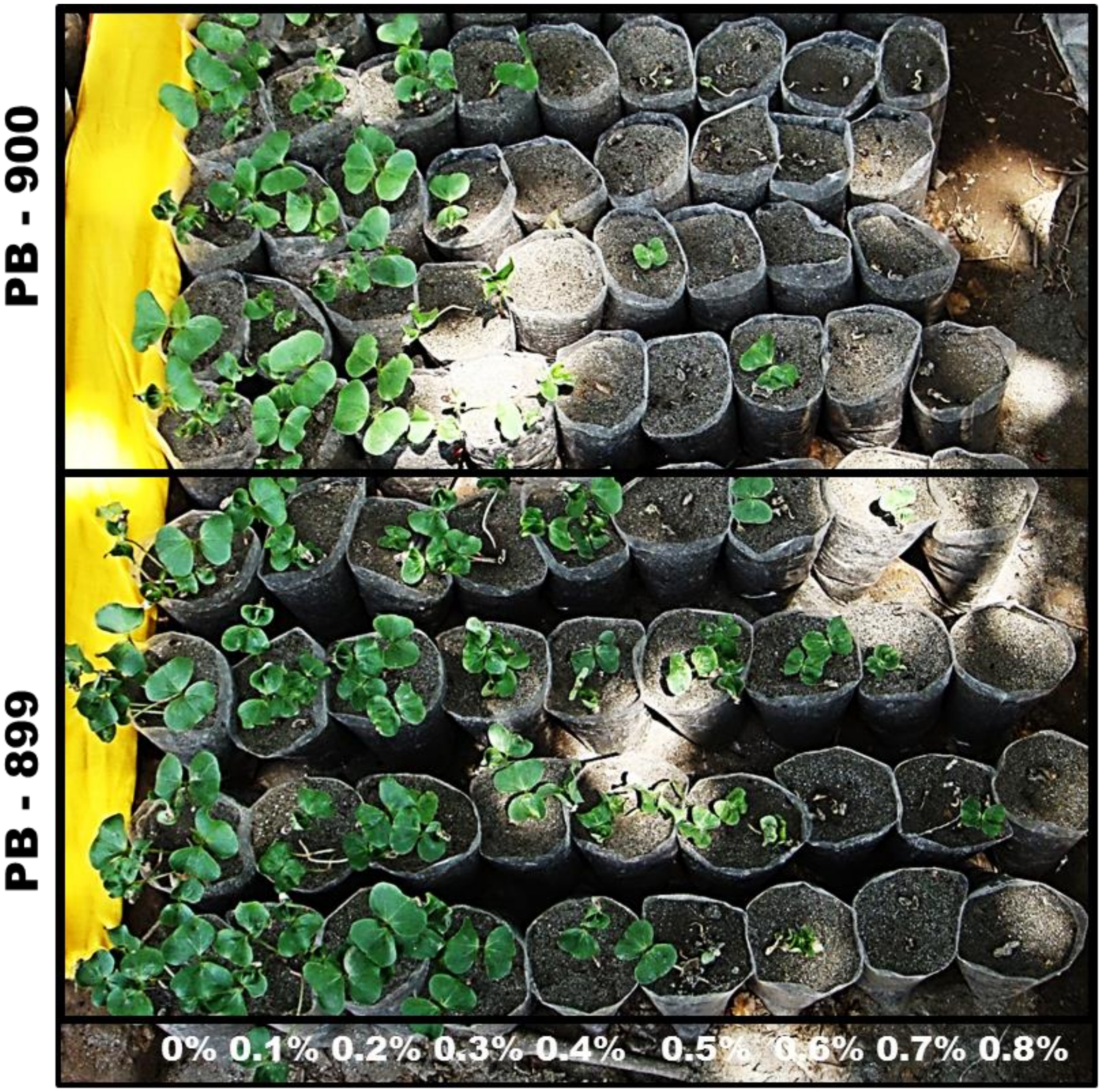

Supplementary Figure 1. Germination percentage test to calculate EMS LD_{50} in "var." PB-899 and PB-900.

A total of 10 cotton seeds were sown per pot. The pots on the far left side in each row contains wild type plants without EMS treatment. EMS treatment was performed in four replicates for each of PB-899 and PB-900. Each plant pot corresponding to dose percentage label is a separate replication.

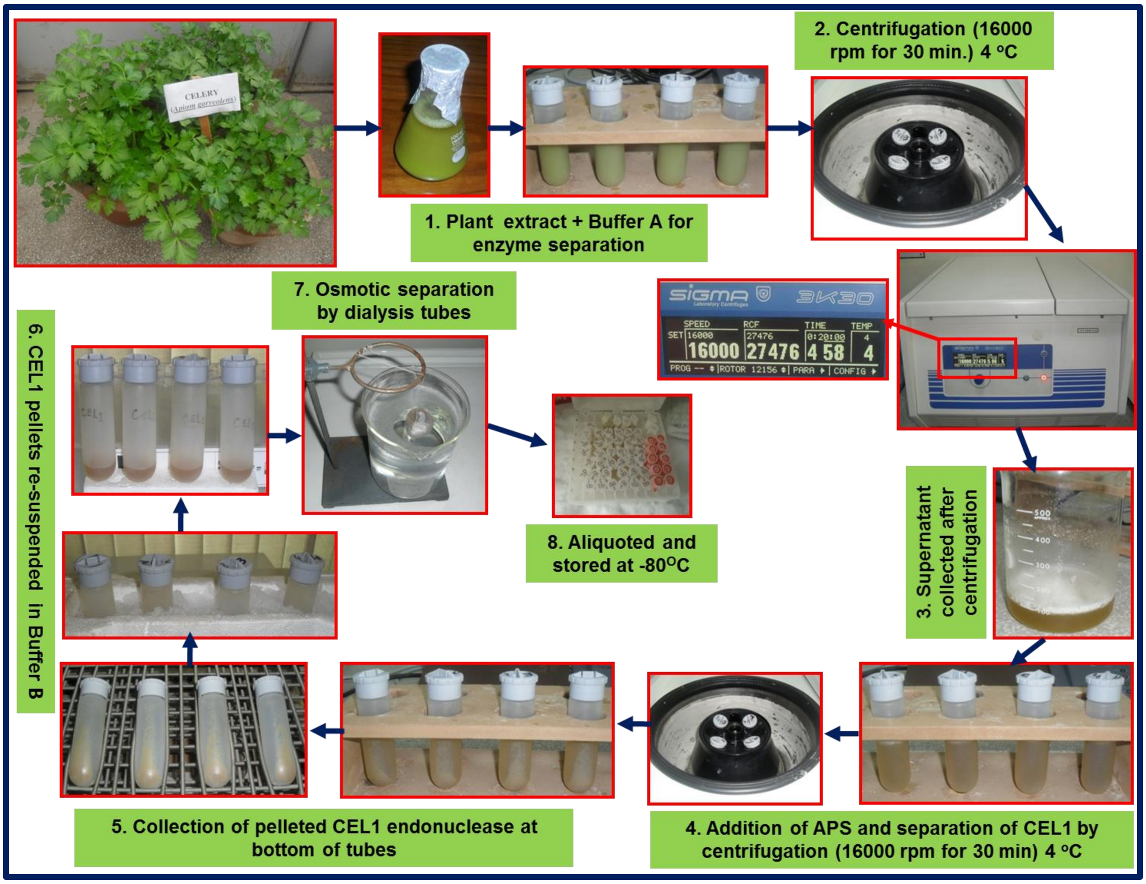

Supplementary Figure 2. Extraction methodology of CEL1 mismatch cleavage endonuclease.

CEL1 extraction setup optimized at the Plant Genetic Resource Lab, department of Plant Breeding and Genetics (PBG), Centre of Agricultural Biochemistry and Biotechnology (CABB), University of Agriculture, Faisalabad. Buffer A in step 1 was prepared in celery juice and formulated as 0.1 M Tris-HCL, pH 7.7, 100 µM PMSF. APS in step 4 stands for ammonium persulfate and used to bring supernatant from 25 % to 55 %. Buffer B in step 6 was prepared in deionized water and formulated as 0.1 M Tris (pH 7.7), 0.5 M KCl, 0.01% Triton X-100 and 100 µM PMSF and used to re-suspend the pellet at the rate of 1-10th volume of initial celery juice extract. (*This figure and all sub-images were taken and designed by Usman Aslam*).

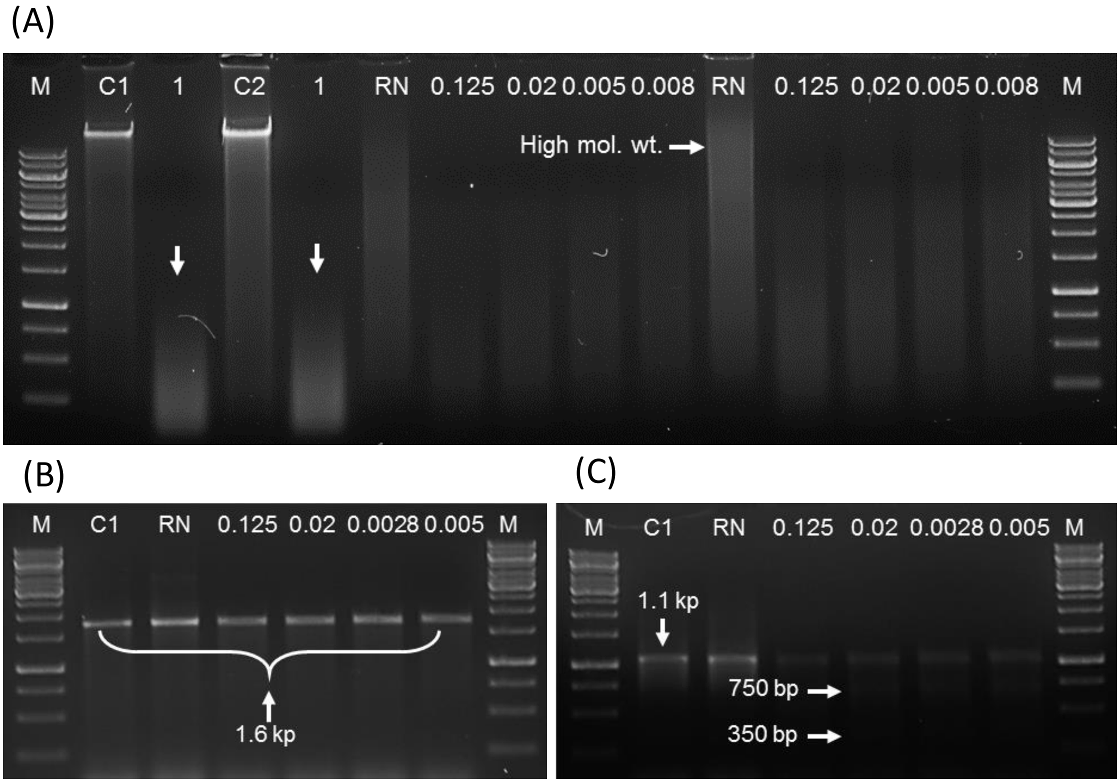

Supplementary Figure 3. Testing and optimization of CEL1 mismatch cleavage activity on cotton genomic DNA and gene specific PCR product.

(A) CEL1 mismatch cleavage assay using genomic DNA. **M:** 1 kb DNA Marker, **C1:** Control untreated genomic DNA of Acala; **1:** Genomic DNA of Acala treated with stock concentration of CEL1 showing typical complete digestion, a property of mismatch cleavage enzymes; **C2:** Control untreated genomic DNA of N-78; **1:** Genomic DNA of N-78 treated with stock concentration of CEL1 showing typical complete digestion, a property of mismatch cleavage enzymes; **RN:** Re-natured DNA; **0.125 to 0.008:** CEL1 treatment of re-natured genomic DNAs with various concentrations showing different rate of digestion. **M:** 1 kb DNA Marker. **(B)** CEL1 mismatch cleavage assay using PCR products without SNPs. **M:** 1 kb DNA Marker, **C1:** *ACTIN* gene; Control untreated PCR amplification product of 1.6 kb. **RN:** Denatured and re-natured PCR product of *ACTIN* gene amplified from two different cotton genotypes, **0.125 to 0.005:** Treatment of re-natured PCR products of two genotypes with different CEL1 enzyme concentrations shown undigested due to absence of heteroduplexes, **M:** 1 kb DNA Marker. **(C)** CEL1 mismatch cleavage assay using PCR product. **M:** 1 kb DNA Marker, **C1:** *NAC1* gene; Control untreated PCR amplification product of 1.1 kb. **RN:** Denatured and re-natured PCR product of *NAC1* gene amplified from two different cotton genotypes, **0.125-0.005:** Treatment of PCR heteroduplexes with different CEL1 concentrations showing cleavage products of 700 and 400 bp.
